# ‘Vaccine as a *cheat sheet*’: a metaphor gone awry on Facebook

**DOI:** 10.3389/fpsyg.2023.1198172

**Published:** 2023-11-20

**Authors:** Elena Negrea-Busuioc

**Affiliations:** National University of Political Studies and Public Administration, Bucharest, Romania

**Keywords:** COVID-19 vaccine metaphor, deliberate metaphor, discourse strategy, conceptual clarification, mockery

## Abstract

COVID-19 vaccine-related conspiracy narratives skyrocketed in social media in the first year of the pandemic. Science communicators have tried to debunk false information as did Vlad Mixich, a Romanian public health expert, who tried to explain on Facebook why the vaccine cannot modify the human DNA. Drawing on the literature on metaphor as a discourse phenomenon, this paper uses a discourse-led approach to metaphor analysis to identify and analyze the metaphors used by Mixich to explain how vaccines work and the mRNA technology underlying the COVID-19 vaccine. A particular metaphor is then given special attention: ‘vaccine as a *cheat sheet*’. The author of the Facebook post seems to use this metaphor deliberately both to clarify vaccine-related information and to mock people susceptible to false information about the vaccine. This paper shows that while the ‘cheating’ metaphor simplifies abstract knowledge and allows the audience to engage with a complex topic, it also has potential to amplify vaccine-related polarization.

## Introduction

On February 15, 2020, at the Munich Security Conference, WHO’s Director-General Tedros Adhanom Ghebreyesus declared that “we are not just fighting a pandemic, we are fighting an infodemic” (WHO, 2020). Thus, in addition to acknowledging the global health threat posed by the coronavirus outbreak, the WHO Director also recognized and warned about the flood of COVID-19 related disinformation, misinformation and conspiracy theories that infiltrated the public discourse. Recent studies have shown that social media have been crucial in polarizing the COVID-19 vaccine discourse (Mønsted and Lehmann, 2022; Ojea Quintana et al., 2022) or in rapidly propagating vaccine-related conspiracy theories (Ginossar et al., 2022) and false information (Carrieri et al., 2019; Scannell et al., 2021). Conspiracy theories on the COVID vaccines (e.g., ‘monitor the world’s population,’ ‘5G implants,’ ‘change human DNA’ as proxies for popular conspiracy theories) are, in essence, global narratives built to appeal to emotions rather than scientific-based knowledge; such narratives may determine greater resilience to persuasion (Scannell et al., 2021) and may contribute to issue and affective polarization (Dan and Dixon, 2021) in society. One of the global conspiracy narratives that had gained the strongest momentum in Romania was that the COVID-19 mRNA vaccines modify the human DNA. Many medical professionals and health communicators have tried to debunk this idea and have engaged in consistent communication efforts to address Romanians’ lack of confidence in the efficiency and safety of the vaccines. An advocate of COVID-19 vaccination, Vlad Mixich^1^ provided a lot of vaccine-related information on social media, including one long Facebook post (791 words)^2^ published on December 29, 2020 (2 days after the official start of the vaccination campaign in Romania). Mixich’s post contains a 6-paragraph detailed explanation of the science behind the use of messenger RNA technology in COVID-19 vaccines, including a paragraph describing a school-related scenario in which the author metaphorically frames the mRNA vaccine as a *cheat sheet* in an attempt to simplify the complex scientific information and debunk the false idea that the mRNA vaccine modifies our DNA. This paper seeks to analyze the use of the ‘vaccine as a *cheat sheet*’ metaphor as a discourse strategy in the naturally occurring online interaction between the author of the Facebook post and his readers.

In addition to conceptual simplification, Mixich’s choice of metaphor seems to have been motivated by a subtle ridicule of people who are not good at science (and who may be more likely to give an ear to vaccine myths), which, in the ensuing Facebook conversation, allows mockery to be interactionally achieved. Drawing on previous work on metaphor as a discourse phenomenon (Wee, 2005a,b; Semino, 2008; Cameron et al., 2009; Steen, 2011, 2015) and on interactional pragmatics approaches to mockery (Drew, 1987; Haugh, 2010, 2014; Arundale, 2010, 2013), this paper aims (a) to examine how the ‘vaccine as a cheat sheet’ metaphor works as a discourse strategy to achieve conceptual clarification, and (b) to show to what extent the use of this metaphor in conjunction with mockery leads to a polarizing conversation, which in turn undermines the basic discourse assumption underpinning objective (a) above, namely that by using this metaphor the author of the Facebook post is engaged in science communication and seeks to debunk public misunderstandings of the mRNA vaccine.

The paper is structured as follows: it starts with a brief review of key approaches to metaphor use in discourse. Then, the methodology and the corpus used are described followed by an examination of conceptual clarification and mockery uses of the ‘vaccine as a cheat sheet’ metaphor. Finally, the overlapping simplification and mockery functions are discussed in the context of the online conversation. It is suggested that the use of this metaphor and the activation of the problematic source domain may contribute to increased polarization around the topic of vaccination in Romania.

### Metaphor as a discourse phenomenon

Metaphors are a precious commodity of human cognition because they allow us to think and talk about complex and abstract ideas in terms of simpler, more concrete concepts (Lakoff and Johnson, 1980). Lakoff and Johnson’s Conceptual Metaphor Theory (CMT) seems to best describe the role of metaphor as a cognitive device as suggested by the rich body of literature that CMT has generated and by the subsequent refinements of the theory. However, CMT has also attracted a good deal of criticism (see Gibbs, 2017, for a thorough review of both supporting evidence and criticism of CMT), much of which targeted the lack of proper attention given by the theory to the communicative aspects of metaphor use in everyday discourse (Cameron, 2002, 2003, 2007; Charteris-Black, 2004; Ritchie, 2004, 2006; Semino, 2008; Steen, 2008, 2015, 2017; Cameron et al., 2009; Musolff and Zinken, 2009; Semino et al., 2013). Previous work on metaphor from a discourse perspective has shed valuable insights into the functions of metaphor use in politics (Charteris-Black, 2004; Chilton, 2004; Musolff, 2010), education (Cameron, 2002, 2003; Deignan et al., 2019), health communication (Semino et al., 2018; Semino, 2021), business communication (Koller, 2004), reconciliation talk (Cameron, 2007), racism and discrimination (Santa Ana, 1999; El Refaie, 2001).

Undoubtedly, COVID-19 vaccines and the vaccination campaigns across the globe are an important topic of the pandemic discourse. As with the pandemic in general, militaristic metaphors were frequently used to explain what vaccines are and how they work in equipping our immune system with the necessary antibodies against the COVID-19 disease. Thus, vaccines are *magic, silver bullets* (Silverman et al., 2020), a *sniper* (Chefneux, 2021), *vital arrows in our epidemiological quiver* or *a weapon* in our *arsenal* to *combat* the virus (Charteris-Black, 2021). However, many source domains other than war have also been used to convey information about the vaccine and vaccination, including fire [individual shot as *a cup of water that can put out a stove fire* (a single case of COVID) and mass vaccination as *a fire hose, −* Silverman et al., 2020], (vaccines as *sprays of flame waylay fire on the move, while also shielding vegetation from the worst of the burn* – Wu, 2021), travel (vaccination as *a train journey*, vaccine shots as *train seats –* Charteris-Black, 2021), *safe delivery and receipt* of the vaccine – Silverman et al., 2020), race (second *sprint* for vaccines, *high-speed* vaccine *rollout* – Charteris-Black, 2021), *awarding the gold medal* to countries for purchasing the vaccines – Chefneux, 2021), chess (vaccine supply and distribution as a *chess game* – Silverman et al., 2020), fairy tales (vaccine as *Prince Charming* – Chefneux, 2021).

Given the novelty and originality of the ‘cheating’ metaphor, this paper aims to provide a detailed examination of its function as a discursive strategy in an asynchronous Facebook interaction. Furthermore, this metaphor is an alternative to warfare rhetoric that was largely (ab)used during the pandemic (Olza et al., 2021), although some scholars doubted the aptness of war metaphors to talk about the pandemic (Semino, 2021).

### Metaphor and conceptual clarification

In science communication, authors (e.g., scientists, journalists, educators, pundits) oftentimes build their own metaphor sources instead of drawing on conventionalized, pre-existing analogies when describing and explaining abstract concepts. When constructing a metaphorical source, the speaker foregrounds that source to capture reader’s attention so that the latter understands the target via a source with which it shares relevant structural correspondences (Wee, 2005a,b). Examples include: Kosslyn and Koenig’s neural network computation as ‘intertwined octopi’ (Wee, 2005a), Dennett’s consciousness as ‘fame in the brain’ (Semino, 2008), the Human Genome as ‘the book of life’ (Hellsten, 2005) or as ‘a draft’ (Bostanci, 2010). Metaphors with constructed sources tend to fulfill specific discursive goals when processed as class inclusion (e.g., blurring the distinction between the source and the target: Wee, 2005b, p. 220) or correspondence (e.g., systematically mapping relations in the source onto the target: Wee, 2005b, p. 221) models. Furthermore, Wee suggests that class inclusion and correspondence models are two different types of “metaphor strategies” (Wee, 2005b, p. 220) used for different discourse purposes. In science texts, the correspondence model seems to be preferred by authors as their presumed intention is to simplify of complex scientific concepts by drawing explanatory analogies with simpler, more familiar ideas. The metaphor discussed in this paper involves a constructed source (*cheating*) that is mapped onto a target (*mRNA vaccine*) in an elaborate school-related metaphorical scenario in which elements of the source domain are recontextualized through correspondences between source and target (Wee, 2005b, p. 371).

Fully acknowledging the limitations of CMT regarding metaphor in discourse, Steen (2008) proposes a three-dimensional model that accounts for undervalued aspects of metaphor use in communication in addition to its conceptual and linguistic dimensions. Steen has expanded this model into a more sophisticated theory known as Deliberate Metaphor Theory – DMT (Steen, 2011, 2015, 2017) that distinguishes between deliberate and non-deliberate metaphors, where only the former are used *as* metaphors to achieve specific communication goals. Despite criticism (Gibbs, 2011, 2015; Gibbs and Chen, 2017) directed particularly at the idea that deliberate metaphors are a special class distinct from other forms of metaphoric language, DMT offers interesting points about the “express use in production and/or reception” (Steen, 2008, p. 223) of a source domain to (creatively) bring a new perspective on the target of a metaphor. Deliberate metaphors, unlike non-deliberate metaphors, are “a matter of communication between language users” (Reijnierse et al., 2020, p. 14) because they help the audience recognize the communicative goal that the speaker might have had when they used a specific metaphor.

Mixich’s Facebook post analyzed in this paper is about complex scientific knowledge – the technology underlying a mRNA vaccine – and by using the ‘vaccine as a cheat sheet’ metaphor the author is seemingly concerned with conceptual clarification. Mixich even signals to his readers that he uses the metaphorical correspondence between the vaccine and an unconventional, but presumably more familiar domain – a student cheating in an exam – to simplify the complicated technical idea by translating it into more familiar terms.

### Metaphor, mockery, and conversation

One of the core ideas of discursive approaches to metaphor is that it may be a dynamic phenomenon emerging from interaction (Cameron, 2003, 2007; Gibbs and Cameron, 2008; Cameron et al., 2009) whose interpretation in conversation is a matter of negotiating, calibrating, and maintaining common ground (Ritchie, 2004, 2010). Consistent with Cameron’s discourse dynamic approach to metaphor use, Ritchie’s (2010) work on metaphors in conversation sheds light on the power of metaphors to build up stories and to stimulate participants to engage in collaborative storytelling. Metaphors are constantly transformed, elaborated, co-created in conversational interaction for the benefits of their relational properties, including bonding through humor, rather than for their informative of persuasive functions (Ritchie and Schell, 2009; Ritchie, 2010). In the analyzed Facebook post, the metaphor ‘vaccine as a cheat sheet’ seems to serve both as a conceptual clarification tool and as a springboard for mockery. Arguably, the metaphor is a crucial component of a mocking remark targeting ignorant Romanians who might be more familiar with ‘cheating’ in school exams.

From an interactional pragmatics point of view, mockery is a “form of interactional practice” (Haugh, 2014, p. 78) that requires the speaker to frame some remark as humorous or non-serious and the participants to recognize and treat it as such in their subsequent responses. Jocular (non-serious) mockery is usually signaled in discourse, either by non-verbal cues or by linguistic markers (Drew, 1987; Haugh, 2010, 2014). Mixich straightforwardly indicates that the use of the ‘cheating’ metaphor is targeted at people “who did not have good grades in biology in high school” and, therefore, might need a “simpler version of the explanation.” Another indication of mockery comes at the end of the paragraph where Mixich ostensibly shows his preference for people who did well in biology as opposed to those who did not. Since the conversation is carried out on Facebook, the author of the post also used emoji to signal the jocularity of his remarks.

## Corpus and method

The corpus of this study consists of Vlad Mixich’s Facebook post published on December 29, 2020, 2 days after the start of the COVID-19 vaccination in Romania, and the comments that it generated, since the latter are an indication of the audience’s high engagement with the content. The Facebook post sparked 722 comments in total, however only 121 were analyzed here, in which the paragraph comprising the ‘cheating’ description is explicitly mentioned. Comments containing only emoji, tags, memes, or links were excluded from the corpus. Cameron et al.’s (2009) discourse-led approach to metaphor analysis was used to identify and group the vaccine metaphors in the corpus. The method consists of identifying linguistic metaphors and indicating their vehicle terms, based on the acknowledgment of the incongruity (inconsistency) between the contextual meaning of the vehicle term and a more basic (i.e., a more concrete, more precise, see Pragglejaz Group, 2007) meaning of the term. Vehicle terms are words or phrases used metaphorically. Each vehicle term is assigned to a vehicle grouping that captures its semantic meaning. Vehicle groupings emerge from data and are used to find patterns of metaphor systematicity (see Appendix Table 1 for a summary of grouping vehicles and systematic metaphors found in the corpus). Systematic metaphors need not necessarily be conceptual metaphors (Cameron, 2008), although they could potentially allude to conceptual domains shared by discourse participants.

The coding of vehicles and linguistic metaphors was carried out manually by the author and another colleague. The Facebook post was examined separately by each coder and then in discussion by both coders in order to reach agreement. All problematic cases were recorded in the Excel file and discussed one by one before a decision about marking/non-marking it as metaphor was made to both coders’ satisfaction.

Between March 2020 and March 2022, Vlad Mixich published 33 posts about vaccination and the pandemic on his Facebook page, 4 of which triggered over 500 comments. In this paper, one of the overperforming posts in terms of number of comments is analyzed. The post published on December 29, 2020 and the comments it triggered were selected because of the presence of the ‘vaccine as a cheat sheet’ metaphor. The Facebook post and the comments contained various metaphors, the majority of which involved mappings of features from the warfare source domain onto the target (Appendix Table 1).

## Findings

### Vaccine as a cheat sheet: metaphor and conceptual simplification

Mixich’s Facebook post is quite a long text (791 words, 6 paragraphs) to be published on social media, in which the author aims to explain the technology underlying the mRNA vaccine against COVID-19 to debunk the myth then circulating in Romania and according to which the vaccine modifies the human DNA. mRNA vaccines use genetic material (mRNA created in laboratory) as a blueprint to teach our cells how to make Spike proteins that trigger an immune response in our bodies. mRNA contained by the vaccine does not enter the nucleus of the cell (where DNA is located) and does not alter or modify the human genes.^3^

In the middle of the FB post,^4^ Mixich suggests that readers view the target (the mRNA vaccine) as a ‘cheat sheet’ used by a student to cheat in a biology exam and builds a 1-paragraph long metaphorical scenario in which he unveils correspondences between how a mRNA vaccine works and cheating in an exam. My aim here is to explore the communicative and pragmatic implications of the use of the ‘vaccine as a cheat sheet’ metaphor in the socio-discursive context in which it has emerged.

There is a simpler version of the explanation, for those who didn’t have good grades in biology in high school. A pupil has a biology exam, but he’s not in the mood for studying. However, he has an elder sister who is a hardworking student and who had to take the same biology exam in the previous year. The sister went diligently to the library (the cellular nucleus in which our DNA is located) and summarized the biology textbook. The sister lends her handouts (the mRNA of the cell) to her lil’ brother who can’t bring them to the exam. They are too large to be used to cheat in the exam and the teach [noun informal ‘teacher’] could catch him. Therefore, he breaks them down into many little thin cheat sheets (this is the mRNA synthetized by researchers and contained by the vaccine) which he uses in the exam. With these cheat sheets our pupil manages to get a 7 (out of 10) to the exam and to pass the class (that is to increase his immunity against the coronavirus up to a satisfactory level). But this does not mean that he actually went to the library and opened the original textbooks (our DNA).

For those who did well in biology (without cheat sheets 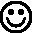), some recap below and a more detailed explanation.

**Figure 1:**
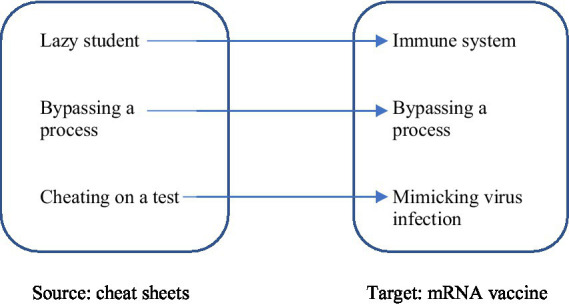
Schematic representation of the correspondence strategy.

This is an elaborate school exam scenario involving students, libraries, studying hard but also cheating. This paragraph breaks with the rest of the text in terms of the style used, high frequency of parenthetical expressions, and the use of emoji. The mRNA vaccine is described as a ‘cheat sheet’ that a lazy student uses to cheat on a biology test. Most likely, the correspondence strategy is used to communicate how the source (cheat sheet) models the target (mRNA vaccine). The correspondences between the target (mRNA vaccine) and the source (cheat sheets) are marked in parentheticals to instruct the reader on how exactly the metaphor should be understood and how the mappings between the source and the target should be established. Figure 1 illustrates the correspondence strategy in the ‘vaccine as a cheat sheet’ metaphor.

Cheating as bypassing the learning process is mapped onto mRNA vaccine as bypassing the infection process by providing the immune system with instructions (cheat sheets) on how to make a protein that triggers an immune response. The mRNA vaccine supplies the immune system with the cheat sheets (copied instructions) needed to manufacture the protein necessary to recognize the virus. Like a cheating student, the vaccine bypasses the learning process by not entering the library (cellular nucleus) and touching the original textbooks (our DNA). Mixich’s communicative goal is to simplify complex scientific knowledge about the vaccine and debunk misinformation that the mRNA enters the cellular nucleus and modifies the DNA of a vaccinated individual.

However, for some elements the metaphorical mappings onto the target are less overt in the absence of Mixich’s instructions. Left explicitly unguided, readers might infer correspondences between ‘lazy little brother who cheats in the biology exam’ and ‘the immune system’ or between ‘biology exam’ and ‘the COVID-19 disease’, though the mapping of ‘diligent elder sister’ (scientists perhaps?) onto some feature of the target domain seems to elude identification.

### Vaccine as a cheat sheet: mocking with metaphor

The choice of the metaphor ‘vaccine as a *cheat sheet*’ seems to have been motivated by more than merely conceptual clarification. Arguably, Mixich constructed the metaphorical source (‘cheating’) and the school scenario to mock Romanians who may not have done so well in school at basic biology and, thus, may be more prone to fall for conspiracy theories according to which the mRNA vaccine modifies the human DNA. Pointing fingers at people who do not know basic biology (i.e., the targets of the mockery) does not entail a shift in the topic of the Facebook post; there is, however, a shift in the framing of the topic from an initially metaphorical explanatory frame (i.e., mRNA vaccine works as cheating in an exam) to a provocative frame (i.e., plagiarism, academic dishonesty). This frame-shifting triggers humorous effects (Ritchie, 2005), which take over the communicative effects associated with the explanation, such as acquiring new information, or understanding how a scientific phenomenon works. The humor and playfulness of mockery is recognized and extended in interaction by both Mixich and people who comment to his post.

The source of the metaphor – cheating in an exam – raises the salience of a socially disapproved behavior – plagiarism – and, thus, establishes common ground between the participants to the interaction on Facebook. People who may have resorted to cheating in school to obtain advantages (good grades, school degrees) tend to be less concerned with education, scientific facts, etc., and, therefore, they may be more susceptible to (dis-) misinformation about the vaccine. Mixich’s mockery targets ignorant Romanians who might better understand the functioning of the vaccine via the ‘cheat sheet’ analogy because cheating may be familiar to them.

### Vaccine as a cheat sheet: readers’ reactions to conceptual clarification and mockery

Has the use of ‘vaccine as cheat sheet’ metaphor simplified complex knowledge about the mRNA technology and helped dismiss misinformation about the vaccine modifying the human DNA? It is virtually impossible to rely exclusively on discourse to ascertain the relationship between the use of a metaphor and a change in people’s behavior. However, from the reactions that Mixich’s Facebook post has generated, one could gain access to what people think about the power of the ‘cheating’ metaphor to facilitate their understanding of the mRNA vaccine.

Many of the comments expressed readers’ appreciation of the explanation which was considered “excellent,” “very clear” and “accessible for everyone.” Very few of the comments echoed the ‘cheat sheet’ metaphor which could indicate that the choice of a constructed source (‘cheating in an exam’) may have simplified the target (mRNA technology) and, thus, may have led to conceptual clarification. One commentator expanded the metaphor via reorganization/reinterpretation of source-related information presented in the Facebook post:

So, the sister who studied in the library (caught the disease) got a better grade (antibodies) than her brother who used the cheat sheets (got the vaccine)! (Comment 57)

Mixich uses ‘library’ metaphorically to describe *the cellular nucleus in which our DNA is located*, but the participant to the conversation adds to conceptual simplification the use of ‘studied in the library’ as a metaphor for *catching the disease* (getting COVID-19) to better emphasize the metaphorical association between ‘using cheat sheets’ and *getting the vaccine*. However, this comment also includes a reference to developing antibodies and acquiring immunity to the disease (absent from the Facebook post), which is explained in a way that is coherent with the cheating metaphorical scenario as “getting a better grade.” It is implied that people who get the disease develop more antibodies than those who get vaccinated.

However, one of the earliest commentators rejected Mixich’s metaphorical explanation on the grounds of it being “too complicated,” which irritated the Romanian health expert who replied: “this is not a battle of explanations!” The follower proposed an alternative metaphor (also involving a constructed source) that they deemed easier to understand and better resonating with the public. ‘Vaccine as a foreman’ metaphor mapped the mRNA vaccine onto a *construction foreman who goes to a DIY store and asks for specific materials*. The commentator builds a DIY store scenario to draw correspondences between what he considers to be a common experience (i.e., shopping in a DIY store) and the complicated technology of the mRNA vaccine.

Do you know those DIY stores where foremen dressed in overalls go to the building department and ask for boards that the employees cut to be used for building a fence, or they ask for OSB boards that the employees need to cut in a certain manner to be used for building shelves … Well, similarly, the vaccine disguises itself as a foreman with overalls so that the cell does not realize that this guy with a shopping list for materials and with instruction is from another planet. So, the employees cut the OSB boards for him according to the required shape and, surprise, the result is a spike-shaped board. (Comment 3)

Another comment that questioned the power of the ‘cheating’ metaphor to simplify complex science underlying mRNA technology reads:

If the brother cheats using his elder sister’s reading notes, why does he become allergic to cheating? (Comment 89)

Here the implication of the metaphor extension is that people are reluctant to vaccinate fearing that they might develop allergic reactions to the vaccine. The metaphor might not help people understand how the vaccine works since it seems to fail to explain why some people become allergic to the vaccine.

By far, the majority of the 121 analyzed comments echoed the mockery implied by Mixich when using the ‘cheating’ metaphor together with mockery targeting Romanians who cheated in school. Mixich’s followers reacted to the mockery either by agreeing to the intended ridicule or by countering the mockery as inappropriate in the context of the interaction (i.e., simplifying complex information about the mRNA vaccine to debunk conspiracy theory narratives). Drawing on Haugh’s (2014, p. 95) model of interactional dynamics of jocular mockery, Table 1 comprises examples of how participants to the Facebook conversation responded to mockery entailed by the ‘vaccine as a cheat sheet’ metaphor used by Mixich as a discourse strategy aimed primarily at simplification and conceptual clarification.

**Table 1:** Response strategies to mockery entailed by the ‘vaccine as a cheat sheet’ metaphor.

Mockery remark (Mixich’s Facebook post)	Response strategy (comments)	Examples
*Vaccine as a cheat sheet* is a simpler explanation for those who did not have good grades in biology	Agreeing with the mockery	Very good explanation! However, those whom you target will never read such a long post…I’m sorry to say it, but it’s the least we could learn from Trump. This is explained in vain. Romanians did not learn genetics in high school because it was a tough subject.
	Elaborating	I admire your determination when explaining this, the problem is that the target does not even know what DNA and RNA mean, to distinguish between them is too much. If you think that our functional illiterates manage to read this text and to understand it, then you are a bit naïve.
	Countering	Since it is the first approved vaccine based on this technology those who got an A+ in biology are in the same boat as the rest. This is for the physicians on the net, all highly educated. Nice example with the student who used cheat sheets. You forgot to mention that that student passed the entrance exam to the medical school in the same way and is now a physician in a hospital.
	Rejecting	By segmenting the audience into smart and stupid people you’ll get nothing. How do you persuade a skeptic by telling them that they are stupid, that it’s not that hard if you think about it a little bit, that you are the smartest and you are going to enlighten them. I like the explanation, but I believe that the comparison to the school and cheat sheets is not quite appropriate to persuade a layperson, they may feel scoffed.

The metaphor ‘vaccine is a cheat sheet’ links the source and the target in ways that go beyond conceptual clarification by evoking features of the source domain that are morally condemned, i.e., plagiarism. Amplified by public accusations against high-level governmental and political figures, plagiarism has become a polarizing topic in Romanian society, dividing Romanians into those who despise people who disregard the importance of (academic) integrity and build their careers on a questionable educational background, on the one hand, and those who sympathize with some people’s ability to succeed in politics and in life, in general, despite not taking education seriously, on the other.

Agreeing with mockery and elaborating it further seem to be commentators’ preferred strategies to interact with the mockery, possibly due to the negative entailments of the ‘vaccine as a cheat sheet’ metaphor. Arguably, the implication is that metaphorical explanation would simplify information for people who do not know basic biology because they have not been too diligent in school but who may be familiar with cheating on a (biology) test. Sometimes, the mockery implied by Mixich’s use of the ‘cheating’ metaphor is extended by the commentators to ad-hominem arguments (see the two threads below) against contestations of the explanatory power of the metaphor itself.

[…] this [paragraph] does not seem too clear for the public, maybe supplemental information could be added in parenthesis. (Comment 13)

After that paragraph, he explains it in an accessible manner for everyone belonging to the two categories, those with poor grades and those with great grades in biology. If you read the text, you’ll find an explanation that fits you. (Thread to comment 13)

Given your knowledge and expertise, I’d expect that you believe the Earth is flat. He [Mixich] explains it in a very accessible manner and if you had known a little bit of biology, you would have realized how stupid you are. (Thread to comment 54)

Arguably, the efficacy of the ‘vaccine as a cheat sheet’ metaphor as a discursive strategy aimed at conceptual simplification seemed to have been undermined, to some extent, by using the metaphor in conjunction with mockery. Many of Mixich’s followers reacted to the mockery while failing (or simply ignoring) to assess the explanatory power of the metaphor to simplify complex knowledge about the mRNA technology. It is not clear how the metaphor may have contributed to facilitating their understanding of the mRNA vaccine, since their reactions were directed at the mockery associated with the use of this metaphor.

## Discussion

This paper discusses a metaphor for the COVID-19 vaccine used in an asynchronous Facebook interaction and seeks to provide a thorough analysis of the role that this metaphor plays in making complex scientific information accessible to lay audiences, and in debunking false narratives about the mRNA vaccine. While metaphors’ role in doing science (as ‘theory-constitutive’; see Boyd, 1993) may be subject to contestation (Bostanci, 2010; Taylor and Dewsbury, 2018), their role in promoting science and mediating scientific knowledge for public understanding is widely acknowledged (Väliverronen and Hellsten, 2002; Semino, 2008). Metaphors facilitate the interaction between scientists, science communicators and the public, which is paramount in science communication (Burns et al., 2003). Sometimes, the communicative goal is favored over scientific accuracy (Armon, 2017) when choosing a metaphor to simplify complex scientific knowledge and communicating it to a large audience. This seems to be the case of the metaphor ‘vaccine as a cheat sheet’ used by Vlad Mixich, a Romanian health expert, on Facebook to simplify the complex, abstract mRNA technology and to debunk false information about the vaccine. Mixich’s choice of the ‘cheating’ metaphor to describe how the mRNA vaccine works seems to be motivated by conceptual clarification. His communicative goal is recognizable both in his post, where his intention to provide a “simpler explanation” is announced at the beginning of the paragraph containing the metaphor, and in his readers’ comments which either extend the metaphor or contest it. Mixich constructs the ‘cheating’ source to explain the virus-mimicking aspect of the mRNA vaccine by an analogy with what he considers to be a (quite) familiar concept to his many Romanians, namely cheating in school.

Science communicators often ‘craft’ their sources (Wee, 2005a) when explaining abstract science concept for the purpose of conceptual simplification. According to Wee (2005a,b), a constructed metaphorical source allows the author to highlight the structural similarities that the source shares with the target – the scientific concept. Mixich, however, seems to focus on functional similarities between the source (cheating) and the target (mRNA vaccine). The vaccine functions like cheating: it does not enter the cell nucleus (hence, cannot modify the human DNA), just like cheaters do not go to the library to study before an exam. Another similarity between the mRNA synthetized in the vaccine and cheat sheets evoked by the metaphor: both are discarded after they fulfill their function (e.g., help a student pass an exam and help the immune system produce the Spike protein, respectively).

Arguably, this unusual, perspective-changing way of talking about mRNA vaccines may have been deliberately used by Mixich to fulfill a specific communication goal (Steen, 2008, 2017). By signaling the metaphor and drawing attention to the source domain he uses, Mixich seeks to make sure that conceptual clarification is achieved. Comments to the post suggest that the metaphor is both acknowledged (and extended) and contested. Extensions of the metaphorical meaning, albeit not necessarily contributing to further vaccine-related knowledge simplification (see examples containing references to better grades and allergies discussed in the previous section), indicate a validation of ‘vaccine as cheat sheets’ metaphor’s communicative role in interaction. However, while recognizing the author’s communicative intention, one of the first commentators contested the metaphor used to fulfill this intention. They challenged Mixich’s metaphor’s communicative power because it is “too complicated” and proposed an alternative explanation “for everyone to understand”: ‘vaccine as a foreman’ (see Table 1). The metaphor “vaccine as a cheat sheet’ seems to be resisted in interaction not necessarily for its lack of explanatory power (Gibbs and Siman, 2021) but for its failure to fulfill a communicative goal, namely, to simplify complex knowledge. Mixich seemed vexed at the rejection of his metaphor’s communicative power and defensively reacted to the contester by replying to their comment that it is not a contest of competing explanations.

With the choice of ‘vaccine as a cheat sheet’ metaphor, Mixich’s communicative intentions seems to go beyond merely conceptual clarification. Apparently, not only the author of the Facebook post uses the metaphor to simplify complex information about the vaccine, but he also mocks with this metaphor. Mixich uses the ‘cheating’ metaphorical scenario to mock Romanians who are ignorant and, thus, less suspicious of false information about the mRNA vaccine spread online. Thus, with the same “cheating’ metaphor, Mixich pragmatically intends to achieve conceptual simplification *and* mockery, which, as claimed in this paper, may not be the best strategy to be used in an online interaction that gives people the opportunity to respond (even asynchronically). The metaphor is used to simplify complex knowledge about the mRNA technology while implicitly mocking the target audience presumably in need of vaccine-related conceptual simplification. The combination of mockery and clarification makes Mixich’s choice of metaphor a questionable contribution to public understanding of vaccine-related science.

The jocular mockery intended by Mixich is signaled via language and emoji and recognized as such by participants to interaction who respond to the mocking remarks. Reactions to the mocking remarks include agreement with, elaboration, countering, and rejection of the mockery (see Table 1). Mixich’s commentators maintain the mockery with some of them adding an aggressive facet to the ridicule at the expense of non-serious playfulness, thus shifting from mockery to putdown humor directed at the target (Dynel, 2008; Haugh, 2010; Taylor, 2015). The playfulness and funniness in cheating metaphor-informed elaborations of Mixich’s mockery of some Romanians who need a simpler explanation seems to be surpassed to a certain degree by (ostensible) malice toward the target (see Dynel, 2008 on the aggressive potential of mockery and teasing).

Admittedly, it is impossible to tell from the textual analysis of the comments whether the ‘cheating’ metaphor has been understood (e.g., comprehended, recognized, interpreted, appreciated, see Gibbs, 1992) by Mixich’s followers as a metaphor used to clarify the mRNA technology in the vaccine, despite it being deliberately used (and signaled) by its author. However, the analysis of the comments suggests that some followers recognized Mixich’s intention to mock with the ‘cheating’ metaphor, since they responded either positively (agreement, elaboration) or negatively (countering, rejection) to the mockery. To a certain extent, reacting to the mockery is an indication of metaphor understanding because they seem to have understood at least one of Mixich’s pragmatic intentions when using the metaphor (i.e., to clarify *and to mock*, see Gibbs, 2010).

Perhaps the most intriguing feature of the ‘cheating’ metaphor resides in its potential to polarize, which is visible mainly in agreement and elaboration response strategies. Apparently, recipients display a preference for responding to the cognitive salient and socially biasing frame of plagiarism activated by the metaphor source. Contrary to what the literature suggests (Stivers and Robinson, 2006; Haugh, 2010, 2014), the metaphor-based mockery used by Mixich interrupts the progressivity of interaction by triggering a topic shift: readers focus on plagiarism rather than on ridiculing ignorant people who believe that mRNA vaccine modifies the human DNA. The ‘vaccine as a cheat sheet’ metaphor used to mock seems to undermine Mixich’s discourse agenda – to debunk disinformation about the vaccine by simplifying and clarifying abstract knowledge. Instead, the metaphor activates salient knowledge about plagiarism shared by participants; a new meaning arises that is not necessarily consistent with the agenda: anti-vaxxers who believe in conspiracy theories about vaccines are more likely to be plagiarizers who did not have basic knowledge about biology because they are ignorant, uneducated people.

Thus, as revealed by some of the readers’ comments, Mixich’s use of the ‘vaccine as cheat sheets’ metaphor in conjunction with mockery may lead to a polarizing online conversation which, in turn, may undermine science communication and the demystification of public misunderstanding of the mRNA vaccine. Metaphor’s power to facilitate science communication and public understanding of how the mRNA vaccine works is subject to contestation. Furthermore, Mixich’s use of this metaphor on Facebook to mock may have contributed to widening the gap between supporters and opponents of a highly polarized (and polarizing) topic: vaccination. Thus, while it simplifies complex knowledge related to vaccines and allows the audience (commentators to Mixich’s FB post) to engage with a complex topic, the metaphor may also amplify polarization by fueling anti-vaxxers’ use of argumentation fallacies (e.g., ad-hominem) to disarm vaccination supporters. Undoubtedly, empirical research is needed to put these assumptions to test.

Finally, it is worth emphasizing that it is not my intention to make any claims regarding the efficacy of the ‘vaccine as a cheat sheet’ metaphor in persuading (vaccine-hesitant) Romanians of the safety of the vaccine. This study merely attempts to offer some insights into how this metaphor is used as a discourse strategy to simplify complex scientific information and debunk COVID-19 myths about the mRNA vaccine modifying human DNA.

## Conclusion

Metaphors are complex and powerful discourse phenomena indispensable to science communication and understanding. This study aimed to shed some light on the versatility of metaphor use in a COVID-19 vaccine-related interaction on Facebook. The use of the ‘vaccine as a cheat sheet’ metaphor for conceptual clarification and mockery is analyzed to showcase how the same metaphor is employed to serve two discursive functions concomitantly in an asynchronous online interaction between Vlad Mixich, a Romanian health expert and communicator, and his Facebook readers. Conceptual clarification and mockery seem to overlap during the online interaction, with mockery monopolizing the exchange due to the salience and polarizing potential of the cheating source domain. This study comes with some limitations which could be pursued in future research. A major limitation is the small corpus analyzed, consisting of only one Facebook post and comments that included references to the metaphorical description of the COVID-19 vaccine as a ‘cheat sheet.’ Nonetheless, the study merely aims at providing some insights into how the metaphor is used as a discursive strategy to achieve conceptual clarification and how this role is identified, negotiated, and contested by participants during interaction on Facebook. This analysis does not aim at making claims about the efficacy of the metaphor in persuading people to change their behavior in relation to vaccination.

This study examined asynchronous interaction which affords that (some) readers’ responses to Mixich’s message referenced exclusively the mockery entailments while overshadowing the conceptual simplification carried out using the ‘cheating’ metaphor. Analyzing quasi-synchronous online interaction might help capture a fuller picture of how the communicative functions of metaphor use are negotiated and fulfilled. Furthermore, more research on the role of metaphors in science communication and popularization might reveal how overloading metaphors with overlapping communication functions may result in topic shifts, confuse the audience and (possibly) distract them from an established discourse agenda.

## Data availability statement

The original contributions presented in the study are included in the article/Supplementary material, further inquiries can be directed to the corresponding author.

## Author contributions

The author confirms being the sole contributor of this work and has approved it for publication.

## Footnotes

^1^A Romanian health expert and communicator, Vlad Mixich is fairly known to the Romanian public, due to his active involvement in the popularization of medical science and the promotion of health-related topics both in mainstream and social media (mainly via his blog https://www.mixich.ro and Facebook page https://www.facebook.com/vlad.mixich). He has worked for the Romanian Ministry of Health (former senior advisor to the Minister and former Vice President to the Romanian Agency for Medicine and Medical Devices), the Romanian Health Observatory NGO (former director), and has extensive experience as health policy expert working for the European Commission, the World Bank, and other international organizations.

^2^This post is part of a larger corpus that comprises 33 posts about COVID-19 vaccination published on Facebook by Mixich between March 2020 and March 2022.

^3^See World Health Organization, Centers for Disease Control and Preventions and Romanian Government-sanctioned information and resources about COVID-19 vaccines available at https://www.cdc.gov/coronavirus/2019-ncov/vaccines/different-vaccines/how-they-work.html, https://www.who.int/news-room/feature-stories/detail/the-race-for-a-covid-19-vaccine-explained, https://vaccinare-covid.gov.ro/scurte-clarificari-cu-privire-la-presupusele-efecte-pe-care-vaccinurile-impotriva-covid-19-le-ar-avea-asupra-adn-ului-uman/.

^4^https://www.faceboo‑FoMvVMsJHPAk37Nuf4B5eMeEyduovZc9AyBaj4nXs3sRwDnixDGZl
