# Supplementary material for: ‘Vaccine as a cheat sheet’: a metaphor gone awry on Facebook
Source: Front Psychol. 2023 Nov 20;14:1198172. doi: 10.3389/fpsyg.2023.1198172 (PMC10694614; doi:10.3389/fpsyg.2023.1198172)
Supplement: Supplementary file 1 [file Table_1.docx]

Supplementary Material

**Appendix Table 1. Metaphor vehicles clusters and systematic metaphors found in Mixich’s Facebook post and in comments.**

| **Vehicle domain groupings** | **Systematic metaphors** | **Linguistic metaphors** | **Examples** |
| --- | --- | --- | --- |
| MILITARY  METAPHORS | IMMUNE SYSTEM AS AN ARMY | cannon  bullets  arrows  bludgeon  annihilate | If cannons do not work, then it tries with bullets, arrows, bludgeons, rockets until it finds out what works. (Comment 3) |
|  | VIRUS AS AN ENEMY | destroys  attacks  fight against the virus | Our immune system to efficiently fight the coronavirus (Facebook post)  Come and see what attacks it (Comment 17) |
|  | VACCINE AS A SHIELD | invisible shield | The vaccine is not an invisible shield (Comment 17) |
|  | ANTIBODIES AS SOLDIERS | soldiers  pillbox | They will still enter the body…but there they will find soldiers in pillboxes and ready to shoot. (Comment 17) |
| CRIMINAL ACTIVITY  METAPHORS | IMMUNE SYSTEM AS A POLICE AGENT | criminal record  police force | It issues a criminal record for the intruder (Comment 3)  Looking for the right police force (Comment 17) |
|  | VIRUS AS A BURGLAR | intruder  on the sly  metal picklock  lock-picking kit | The real coronavirus comes on the sly…with its spike protein as a metal picklock (Comment 3)  The immune system knows how to fight it and it destroys its lock-picking kit (Comment 3) |
| PLAGIARISM METAPHORS | VACCINE AS CHEAT SHEET | cheat sheets  get caught | The vaccine is the cheat sheet that your immune system needs. (Comment 6)  the teacher is alert and catches you cheating (Comment 51) |
|  | COVID-19 AS A BIOLOGY EXAM | exam  get a grade | our student manages to get a 7 to the exam (Facebook post 2) |
|  | mRNA AS HANDOUTS | to summarize  handouts | The sister who summarized the biology textbook…. lends him her handouts (Facebook post 2) |
|  | DNA AS A TEXTBOOK | textbook | Open the original textbook (our DNA) (Facebook post) |
|  | CELLS AS LIBRARY | library | The sister went to the library (cellular nucleus where our DNA is found) (Facebook post) |
| BUILDING METAPHORS | VACCINE AS A FOREMAN | foreman dressed in overalls  list of materials | Do you know those DIY stores where foremen dressed in overalls go to the building department and ask for boards that the employees cut to be used for building a fence, or they ask for OSB boards that the employees need to cut in a certain manner to be used for building shelves…Well, similarly, the vaccine disguises itself as a foreman with overalls so that the cell does not realize this guy with a list of materials…(Comment 3) |
|  | IMMUNE SYSTEM AS DIY STORE | DIY employees  OSB boards  building department |  |
|  | PROTEINS AS BRICKS | bricks | Proteins (the bricks of the human body) (Facebook post) |
|  | SPIKE PROTEINE AS HOUSE KEY | house key | Precisely that protein is the key to enter the house (Comment 57) |
| SPORTS METAPHORS | IMMUNE SYSTEM AS AN ATHLETE | to train | The immune system cannot train instantly (Comment 3) |
| ELECTRICITY METAPHORS | VACCINE AS AN ELECTRIC CHARGE | short-circuiting | “short-circuiting” the virus (Comment 52) |
