# Supplementary figures and images for: ‘Vaccine as a cheat sheet’: a metaphor gone awry on Facebook
Source: Front Psychol. 2023 Nov 20;14:1198172. doi: 10.3389/fpsyg.2023.1198172 (PMC10694614; doi:10.3389/fpsyg.2023.1198172)

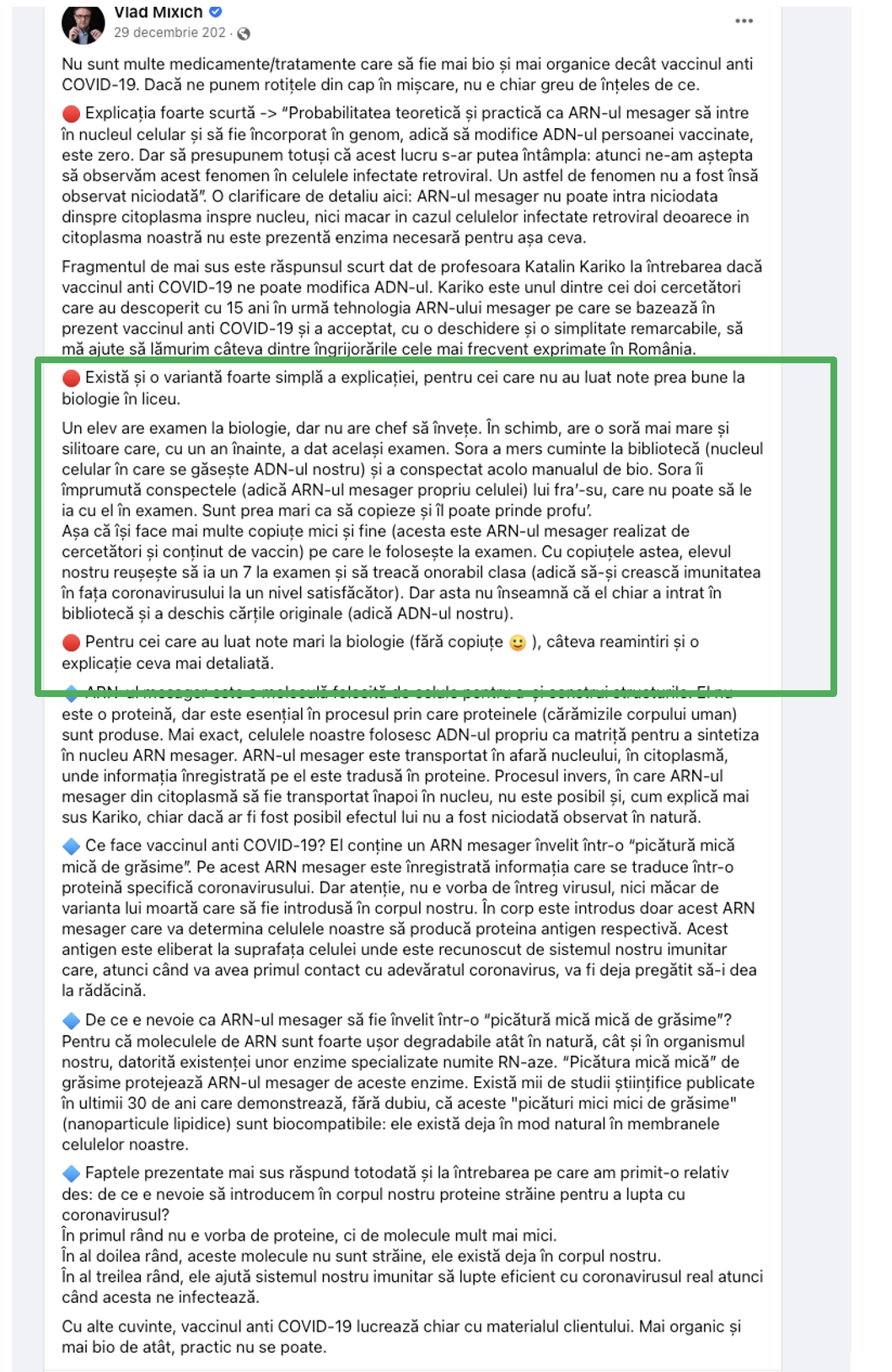

Supplement: Supplementary file 2 [file Image_1.png]
